# Medical News and Miscellany

**Published:** 1889-12

**Authors:** 


### Medical News and Miscellany.

THE NEW ORLEANS POLYCLINIC has an announcement in this issue.

---

MARRIED November 6th inst., at Holly, Houston county, Texas, Dr. R. W. Skipper to Miss Ella S. Lee, both of that city. No cards.

---

REMOVALS.—Dr. T. M. Wilson has removed from Kosse to Thornton.—Dr. H. B. Hill from Gay Hill to Austin.—Dr. J. G. Daniels from Gilmer to Tyler.

---

DIED.—Dr. J. J. Woodson, of Crocket, died last November, and Dr. A. H. Sikes, of Hearne, took corrosive sublimate by mistake for chloral hydrate, and died from the effects of it.

---

WE ARE PROMISED a paper on Puerperal Eclampsia, by Dr. S. H. Stout. Dr. Stout has never lost a case in a practice of fifty years, and the foundation and *sine qua non* of his treatment is the lancet.

---

MARRIED in Austin, December 6, Capt. H. W. Lightfoot, of Paris, Texas, to Miss Etta Imogene, daughter of Dr. T. D. Wooten, of this city. The groom is an ex-State Senator, President of the Texas Bar Association and a law partner of Senator S. B. Maxey,—withal an elegant and distinguished gentleman.

---

THEY VALUE IT.—Messrs. Doliber & Goodale, proprietors of celebrated Mellins food, say: "The fact that we have advertised our Mellins food in DANIEL'S TEXAS MEDICAL JOURNAL for a number of years, and are still using its columns, is evidence that we value it as an advertisiug medium."

---

A SINGULAR MORTALITY in Dr. Wilson's family is recorded in the letter from the doctor, published in the correspondence of this issue. We tender our sympathy to him, and hope our readers who have had experience and success in this dreadful scourge of infants, will give us, and the profession, their views through the JOURNAL. This is an important subject, of practical, everyday interest, and a fitting one for general discussion. We hope to hear from our readers.

---

ATTENTION is called to the new advertisement of the Philadelphia Medical and Surgical Record, a weekly, of sterling worth and acknowledged ability. The Record is perhaps the oldest medical publication in that land of medical journals —Philadelphia, it having been founded by Dr. Butler away back in the fifties or earlier. Subscribe for the Record and DANIEL'S TEXAS MEDICAL JOURNAL and you will have a miue of pleasing information and pleasant reading.

---

THE CENTRAL TEXAS MEDICAL ASSOCIATION will meet in Waco, Tuesday, January, 14, 1890, for which meeting the following progromme has been appointed: "Cerebro-Spinal Meningitis," by Dr. J. C. J. King; "Pneumonia," by Dr. C. T. Young; "Hemorrhoids," by Dr. J. E. Brown; "Cystitis," by Dr. W. C. Blalock; "Diseases Incident to Dentition," by Dr. J. M. Witt. Physicians are cordially invited to be present and present a "volunteer" paper, or report some interesting case from practice. Annual election of officers will take place. Dr. W. O. Wilkes is the secretary.

